# Seasonal timing on a cyclical Earth: Towards a theoretical framework for the evolution of phenology

**DOI:** 10.1371/journal.pbio.3001952

**Published:** 2022-12-27

**Authors:** John S. Park, Eric Post

**Affiliations:** 1 Department of Biology, University of Oxford, Oxford, United Kingdom; 2 Department of Wildlife, Fish, and Conservation Biology, University of California, Davis, Davis, California, United States of America

## Abstract

Phenology refers to the seasonal timing patterns commonly exhibited by life on Earth, from blooming flowers to breeding birds to human agriculture. Climate change is altering abiotic seasonality (e.g., longer summers) and in turn, phenological patterns contained within. However, how phenology should evolve is still an unsolved problem. This problem lies at the crux of predicting future phenological changes that will likely have substantial ecosystem consequences, and more fundamentally, of understanding an undeniably global phenomenon. Most studies have associated proximate environmental variables with phenological responses in case-specific ways, making it difficult to contextualize observations within a general evolutionary framework. We outline the complex but universal ways in which seasonal timing maps onto evolutionary fitness. We borrow lessons from life history theory and evolutionary demography that have benefited from a first principles-based theoretical scaffold. Lastly, we identify key questions for theorists and empiricists to help advance our general understanding of phenology.

## Introduction

Phenology—the seasonal timing of biological events on scales ranging from individual life cycles to global cycles—is a universal feature across plants and animals, from ecosystems (e.g., flowering, emergence, migration) to human systems (e.g., agriculture) [1–3]. Phenology’s ubiquity is perhaps unsurprising: The revolution of the Earth around the sun preceded the origin of life itself and underlay the course of evolution ever since. Thus, phenology is arguably one of the deepest themes in ecology. The rapidly growing interest in phenology over the last few decades has focused on consequences of climate change [2,4]. But explanations of recent phenological changes are typically system specific and focused on empirical cues and responses. This top-down (specific observations first) tendency might, to some extent, be attributed to phenology’s history as more of an amateur natural history interest prior to its recent resurgence in attention with climate change [5]. This recent focus has not yet been matched by developments of a higher-order organization of the principles of phenological selection despite phenology’s global operation and importance [6,7]. Distillation of the first principles of phenological evolution is urgently needed to synthesize and contextualize the large body of disparate reports and explanations of phenological divergence unfolding under climate change. Moving forward, such a theoretical organization will (1) make phenological research more streamlined as new knowledge gets compared against and added to a common conceptual framework; (2) enable baseline predictions of future phenological change even where data to parameterize statistical models are yet insufficient for a system of interest.

**Fig 1:**
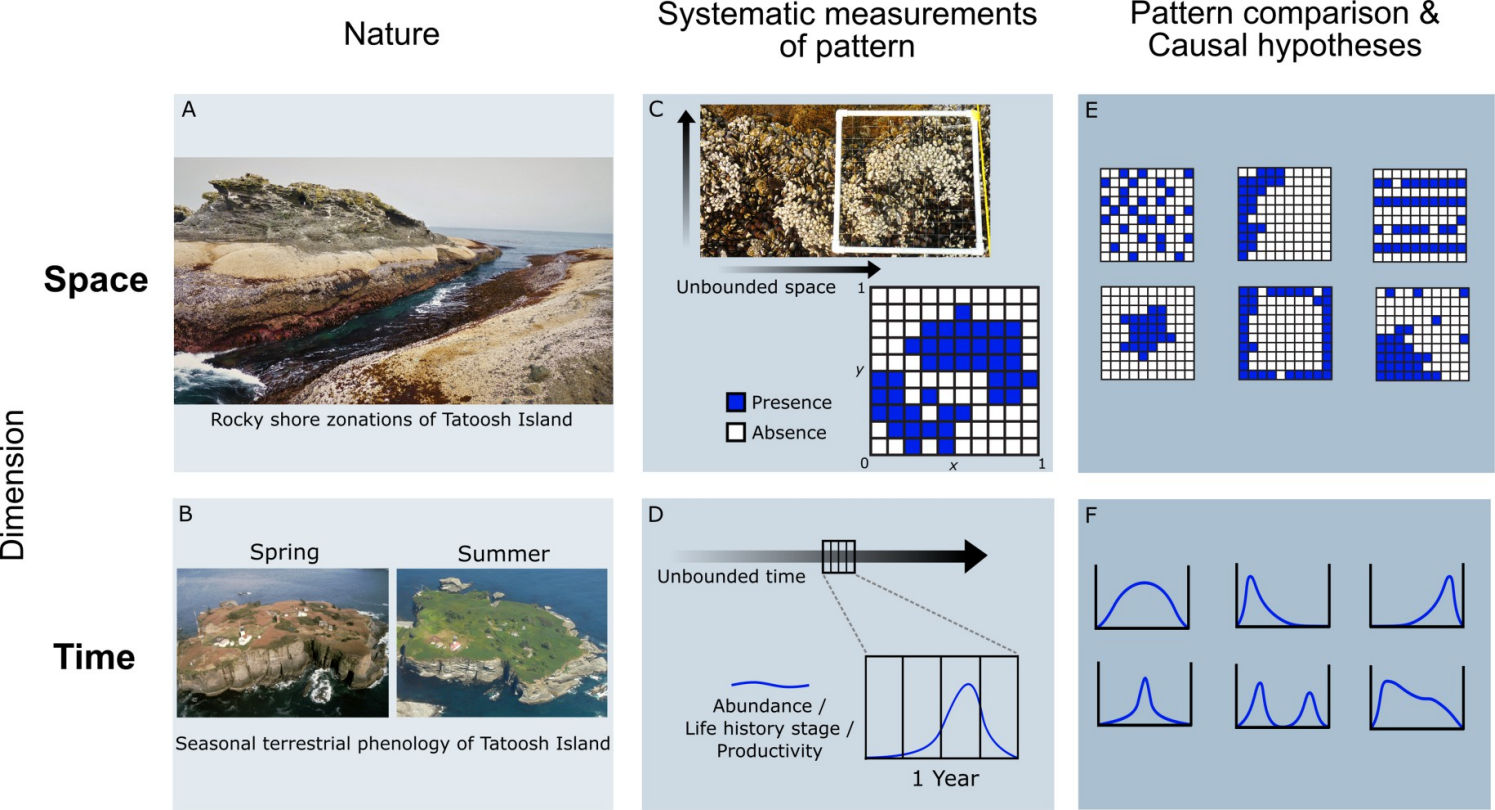
Distribution patterns in finite units of space or time. (A) Repeated patterns across space are often innately obvious to human observers, such as the zonation of rocky shore intertidal communities, even if space in nature is realistically messy and unbounded. (B) Repeated patterns in time, such as seasonal phenology, can be seen over longer observations. (C) Patterns can only be defined and quantitatively measured given finite boundaries. In space, standardized delineations such as transects and grids are commonplace. (D) Just as in space, one must delineate unbounded time into relevant units such as years or climatic growing seasons to quantify time points occupied by phenological expression. (E) Only within standardized arenas of measurement are comparative studies possible and can eco-evolutionary theories of change be developed and tested. (F) Similarly, an explicit view of timing patterns within standardized time windows sets the basis for systematic hypotheses of how phenology is shaped by ecological and evolutionary forces. Photo: J. Timothy Wootton.

Phenology—regardless of scale or system—describes cyclical patterns in the dimension of time (Fig 1). Historically, spatial pattern-thinking has influenced many fundamental theoretical frameworks in ecology and evolution from island biogeography [8] to regional-local community hierarchies [9] to species ranges [10], perhaps due to the immediate obviousness of spatial patterns. However, decades of phenological observations show that there are repeatable and predictable biological patterns in the dimension of time as well. The Earth’s physical environment is structured by temporal cycles, even in comparatively less seasonal environments such as the Tropics in lower latitudes [11]. Such physical cycles bound the time windows for predictable biological dynamics such as seasonal life history events of individual organisms, oscillations in the numbers of individuals in a population expressing such seasonal traits, or in the number of species expressing them.

Climate change influences cyclical timing patterns in 2 main ways. The first is via overall warming [12], e.g., increases in mean annual temperature, which influences rates of biological processes such as development. Studies typically analyze the timing of measurable state transitions such as bud-burst or flowering for plants [3,12–14] and breeding or migration for animals [15–18]. Research on phenological shifts (Box 1) has disproportionately focused on the warming aspect [19] particularly in high-latitude temperate ecosystems, though cloudiness and precipitation seasonality might play a bigger role in lower latitude or arid systems [20–22]. The second is via entire “climatic” growing seasons (e.g., the continuous frost-free period of the year) being extended by earlier springs and later autumns [19,23–27], as well as potentially becoming more variable [23]. The climatic growing season is a period when biological activity is favorable [28] or possible at all. Therefore, changes to the length and predictability of the climatic growing seasons represent an alteration to the arena needed for the unfolding of life cycles, population dynamics, and larger scale ecosystem processes. There is mounting evidence that the warping of the seasonal time window dramatically drives rapid evolution of individual phenological traits [6,28–32] and whole phenophases (Box 1; [33]). That the very temporal arena containing temporal phenological patterns is itself morphing makes the evolutionary process of phenology an ever more complex and intriguing puzzle.

**Box 1. Definitions**

- Phenological shift: directional change in the timing or phase duration of life cycle schedules within the context of geophysically fixed annual oscillations in the environment
- Phenophase: the duration of a categorically distinct phase of a life cycle, such as adolescence or reproductive period
- Proximate phenological causality: system-specific triggers that induce the expression of phenological traits
- Ultimate phenological causality: broad evolutionary forces that influence the seasonal timing of universal life history traits such as birth, growth, reproduction, and death, considering that the timing of each trait contributes to fitness and all are constrained by trade-offs with one another (e.g., earlier birth may incur costs on growth)
- Life history evolution: the evolution of the holistic suite of life cycle traits in the context of the ecology of populations as well as predictable fluctuations in the environment
- Evolutionary demography: selection dynamics that produce, as well as directly result from dynamics in the size and structure (proportions of stages, ages, sizes, or sexes) of populations
- Eco-evolutionary dynamics: the concurrent and reciprocal dynamics of ecological and evolutionary processes where one shapes the context of the other, usually described as a feedback

Perhaps the most unresolved conundrum is that the same change in climatic growing seasons often induces very different phenological shifts between organisms occupying the same habitat in both direction (when) and magnitude (by how much). Discrepancies in shifts are observed among individuals [34], between traits in a single species (e.g., early-life traits shifting more than late-life traits [29,35,36]), as well as among species in ecological communities [37]. Longer climatic growing seasons are not necessarily beneficial nor do they have the same consequence for different populations and species. For example, longer growing seasons have benefited some species (e.g., orchids in Norway [38] or yellow-bellied marmots in the United States [39]), but markedly decreased growth rates of others (e.g., mustard white butterflies in the US [40]). Interestingly, these discrepancies might illuminate an interaction between phenology and demography that makes a wide array of phenological changes more tractable, which is gaining attention [39–45].

In summary: The rapidly growing body of top-down (observations first) studies of phenological change is brimming with contrasting effects and specific explanations, which makes it difficult to generalize the eco-evolutionary links between seasonality change and phenological change across systems [17,46,47]. What is comparatively missing is a bottom-up approach to phenological evolution. Such an approach would first define null expectations and testable hypotheses, which can be used to discern cases where populations might be shifting more than or lagging behind the theoretical expectation. While deviations from null expectations can identify populations that might indeed be failing to track the expectation, deviations can also teach us when, how, and why theoretical assumptions might not hold and how expectations should be updated. Further, a focus on first principles causality might enlighten commonalities between seemingly disparate cases of phenological shifts that differed due to proximate particulars.

In our first section, we argue that a first principles view of phenological evolution starts with the recognition of a simple truth in any system: the fitness consequences of some key phenological traits vary over time within a year. In other words, if any phenological manifestation confers an equivalent consequence for fitness, there would be no discernible phenological pattern around the planet. Drawing analogies from spatially oriented theory, we highlight that discretizing the dimension of time in bounded units enables quantitative conceptualization of how phenological variation maps to variation in fitness. Then, we outline how this variation in fitness produces selection pressures on phenological variation, first at the scale of individuals and populations, and then at the scale of multispecies communities. Our overarching goal is to introduce theorists to the unsolved puzzle of general selective forces acting on phenology around the world and invite empiricists to test those emerging hypotheses to advance cross-system understanding.

### Phenology: Cyclical patterns in the dimension of time

**Fig 2:**
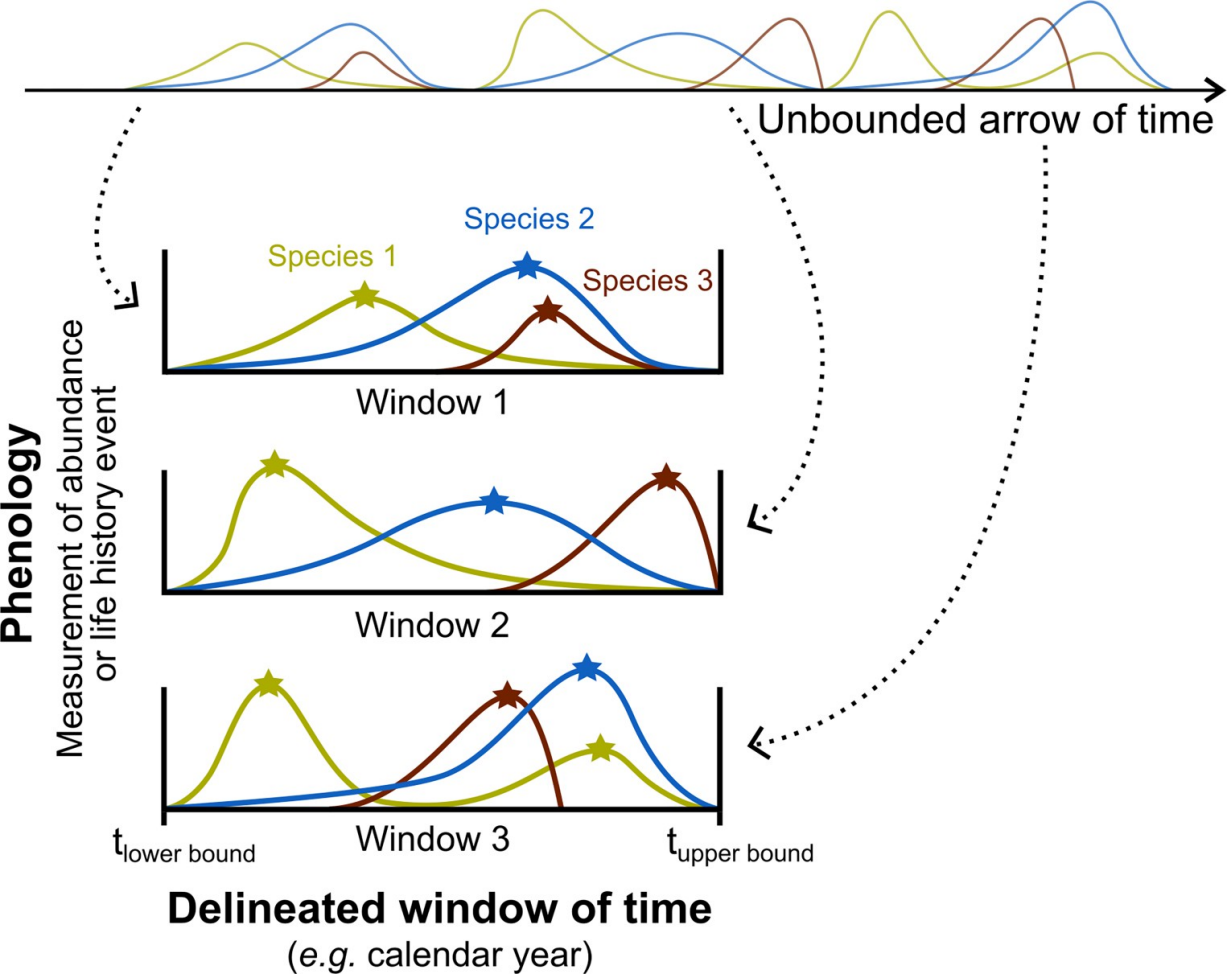
Phenology is a study of repeated patterns of events in the dimension of time. Delimiting the continuous arrow of time into natural units such as years or climatic growing seasons allows observers to compare patterns between cycle periods and quantify change such as phenological shifts. Shapes of seasonal patterns (colored curves), peak dates (location of stars), or number of peaks (e.g., species 1 has 2 peaks in window 3) can be taken to measure change, e.g., in units of days. Systematic quantification and comparisons then provide the necessary groundwork for studying ecological and evolutionary causality.

Patterns, in any dimension, can only be quantified and systematically compared in the context of defined bounds and scales. Drawing analogies from the more familiar spatial dimension helps crystallize this point (Fig 1). Ecologists commonly discretize infinite space into appropriately sized frames for the question at hand even if the chosen scale is imperfect and arbitrary [48]. Consider how we bound nature with transects or grids. We use statistical tools to translate observations within the bounds to an understanding of how and why entities are distributed in space, even though a vector crossing the surface of a spherical planet is in reality infinite. Similarly, while time is in reality boundless, delineation (e.g., the climatic growing season) allows systematic quantification and comparison of phenological timing patterns contained within (Fig 2). In both space and time, some delineations are non-arbitrary and important for biological dynamics, such as islands or habitat boundaries in space and daily or seasonal cycles in time.

Focusing on the temporal bounds that encompass annual patterns might be an important step for first principles theory development because bounds are one of few parameters that are universal. In other words, any system that exhibits cyclical phenological patterns has a beginning and an end to the seasons that constrain the sequence. The relationship between the size of the bounded domains in natural systems—whether in space (area) or time (duration)—and the biological patterns they contain is often complex, and thus inevitably requires mathematical modeling. For example, in space, the size of islands or habitat patches nonlinearly determines biodiversity, distribution, and coexistence patterns contained within that space [8,10,49–52]. Analogously, expansions of the climatic growing season (the “size” of the bounded domain in the time dimension) are associated with complex and often unintuitive phenological pattern changes within and among species within the seasonal window [12,27,53–55], such as change in voltinism (number of generations in a year) [56]. In contrast to the space dimension analogue, theoretical understanding of how changes in the size of the seasonal time window drive phenological change is much more unresolved. Recent theoretical work, however, showed that simple contractions or protractions of the cyclical time window alone can drive diverse and dramatic changes in life history strategies that underpin phenology [42,57].

In theorizing the causality behind any change in temporal patterns, it is also important to keep in mind that cyclical phenological patterns are distinct from emergent “phenomenological cycles” that arise from internal systems dynamics (e.g., predator–prey cycles) or Markovian transition processes (e.g., ecological succession). In contrast, phenological patterns are evolutionarily adaptable strategies that are repeatedly expressed within periods of geophysical environmental cycles (Fig 1; [31,32,58]). As an example for the adaptive nature of phenology, studies using model systems such as *Arabidopsis* show that phenological traits like flowering time are expressed in the laboratory even in the absence of climatic cues characteristic of the natural populations’ localities and can even be mapped to specific genes under selection [59].

Lastly, 2 caveats should be seriously considered when inferring evolutionary causality behind cyclical phenological patterns: temporal contingency in abiotic and biotic dynamics, and scale relativity between life cycles and seasonal cycles.

#### Temporal contingency

**Fig 3:**
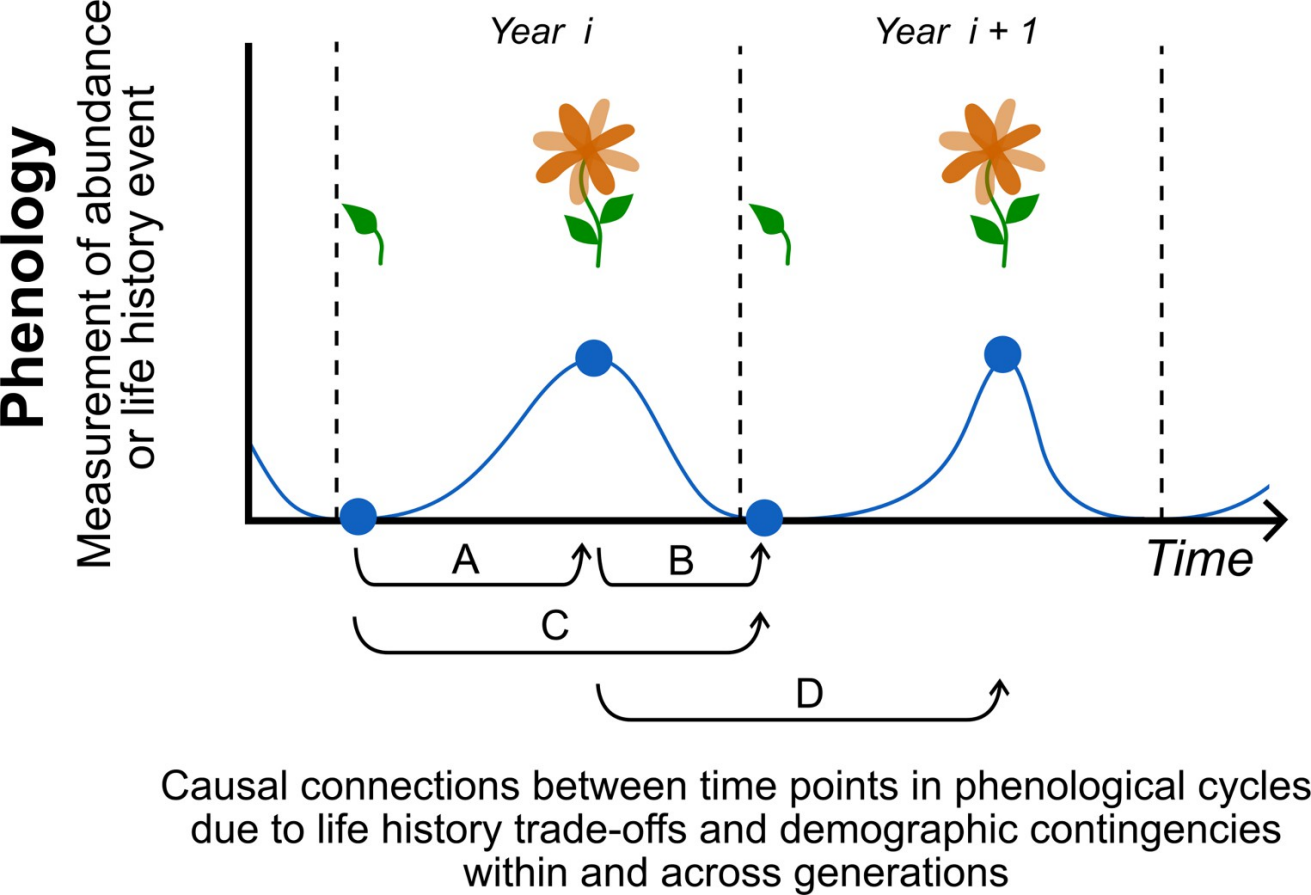
The manner in which phenology evolves at the single-species level requires consideration of trade-offs and temporal contingencies both within an individual’s lifetime and across generations. Phenology treated as a correlative response to meteorological forcing per year overlooks how evolution is shaped by trait covariance and demographic lag effects. Here, we illustrate 4 examples of connections in phenological cycles across 2 adjacent years or generations. Curve shows fluctuations in abundance or event timing (peaks) that are typical representations of phenology. Blue circles paired with pictoral representations of flower development denote points in the life cycle, and arrows indicate causal links between 2 points: (A) Biological functions early in life such as development and growth can be negatively correlated with those later in life such as reproduction. (B) Conversely, reproductive investment in 1 generation can influence the next generation’s offspring performance. Success of the previous generation can also shape the standing genetic variation available for selection in the next generation. (C) In some species, a proportion of offspring or seeds of a population will proceed to development while others enter diapause. These unrealized offspring carry over to subsequent years and influence population dynamics and selection landscapes in the future. (D) If the timing of a phenological trait such as flowering is related to fitness in the context of the environment, selection can shape the frequency distribution and its mean in the population, balanced out by potentially antagonistic forces such as those connections represented by A, B, and C.

Phenological patterns in a given seasonal time window are deeply contingent on past windows, importantly with respect to both the abiotic as well as biological dynamics. Here, the analogy between spatial pattern formation and temporal pattern formation breaks: in space, causation can act bidirectionally in 3 dimensions, but causation is unidirectional (“anisotropic”) in time, from past to future. The anisotropic nature of temporal patterns makes causal influences stronger in time than in space since effects from multiple directions can be counteracted or obfuscated in space [60]. In other words, some or all environmental factors as well as surviving individuals in a biological system in a given time period necessarily had to arise from past time periods. Abiotically speaking, future environments are dependent on past windows, often in an autocorrelative manner with a few dominant time lags. The consequences of temporal autocorrelation in environmental variables such as temperature or food availability have been extensively studied in the contexts of population dynamics [61–63] and life history evolution [64,65]. However, the effect of autocorrelation and temporal contingency on the natural selection of cyclical phenological patterns is much less well understood (but see [66,67]). Biologically speaking, individuals’ future phenological timings are inherently dependent on the individuals’ past allocations and trait expressions (e.g., energy expenditure in early life phenological traits influences the amount of resources individuals need to accrue for subsequent growth, survival, or reproduction, and thus the timing of those transitions, e.g., [54,68]; Fig 3). The population-level distribution of phenotypes is constrained by those whose phenological timing in past windows was compatible with their survival. One fruitful avenue might be to adopt modeling methods developed in evolutionary demography and life history theory that set up the environmental cycles and biological dynamics interactively; models such as adaptive dynamics [54,69] allow the interplay between the 2 types of temporal dependencies to analyze the effect of eco-evolutionary feedback dynamics.

#### Scale relativity

A collection of species that exhibits a repeatable phenological pattern year to year in the same space may consist of strikingly different generation times or activity schedules; hence, those species’ population dynamics actually operate on very different scales of time (e.g., the community of phytoplankton and zooplankton in Lake Washington, USA shows predictably synchronized seasonal temporal patterns but the 2 trophic levels have very different generation times [70]). Annual organisms fit 1 generation within 1 period of an annual cycle, whereas perennial organisms experience multiple periods per generation, and shorter-lived organisms fit multiple generations within the same annual cycle [71]. In space, too, local patterns are influenced by processes larger than the scope of study, which are invisible to the local observer [48]. Analogously, longer processes are invisible to the “brief” observer of natural systems. The key point is that the delineation of time into bounded units is necessary for standardized measurement of the distribution of biological events within time units and development of explanatory theory. The goal is to develop theories that generally explain the widespread phenomenon of seasonal biological rhythms in nature, despite the fact that the scale of seasons means very different things to species with vastly different generation times.

Towards that goal, we ask a general guiding question: How do organisms that live in environments with periodic time windows evolve to utilize nonrandom portions of the windows? We break the question down to 2 key hierarchies: single-species phenological evolution and community interactions that influence multiple coexisting phenologies.

### How can life history and demographic theory help establish first principles of phenological selection?

Phenological timing is typically studied as a variable responding to seasonal transitions in the abiotic environment (e.g., temperature, snow melt, photoperiod, precipitation). Responses to seasonal environmental variables, often involving plastic expressions of traits [2,72], constitute proximate phenological causality (Box 1). Environmental cues often have tractable effects on the timing of trait expression and will continue to be important targets of research as cues will likely continue to shift and become more unpredictable under climate change [2,73,74]. However, proximate investigations often cannot fully explain or predict phenological shifts in many cases; species in the same space experiencing the same change in seasonal cycles often exhibit unexplained variability in phenological shifts [37,47,75–80]. These formerly surprising discrepancies appear to be commonplace and confirm 3 notions: (1) there is of course no single optimal phenological timing, or shift, for all species; (2) there are unexplained evolvability differences between species with respect to their phenologies in response to environmental change; and (3) investigating the correlative trait responses to environmental variables might not be sufficient for understanding the general selective pressures acting on phenological change.

A sense of what constitutes “correct” timing, or the baseline null expectation of how phenological timing should change given some change in the environment, is currently not theoretically generalized. Expectations are often set by intuitions that can arise from system-specific knowledge, e.g., food availability for birds [16]. However, given the geometric nature of population growth and fitness, it is at least theoretically conceivable that a seemingly imperfect matching of phenological timing with respect to some relevant target such as seasonal food peak is actually optimal due to longer-term payoffs [7,81,82]. Post hoc statistical analyses of phenological change with candidate environmental variables cannot easily integrate responses across the lifespan to reveal impacts on lifetime reproductive success, and selection over multiple generations, to offer explanations of ultimate causality (Box 1). Most importantly, a generalized evolutionary framework can allow one to quantify how unexpected an observed phenological shift really was (e.g., statistically unlikely) against null expectations. For example, Park [42] theoretically showed that with small differences in the combinations or magnitudes of life history trade-offs, populations can have dramatic—and directionally opposite—shifts in life histories even when given the same change in environmental seasonality.

Life history theory and evolutionary demography (Box 1) have consistently provided biologists with remarkable causal explanatory power based on simple, species-agnostic frameworks [83,84]. While life history theory has certainly entered the field of phenology [2,28], the likes of the bottom-up theoretical structure that exists in the former discipline has not been established in the latter. Life history and evolutionary demographic theoretical frameworks consider fundamental processes that are universal across organisms such as birth, growth, reproduction, and death. The classic models are free from species-specific assumptions (e.g., [85–88]) and draw broad conclusions about the direction in which life history evolution should proceed if, for example, certain age classes experience selective mortality. The classic models then extend the calculation to the population level by conceptualizing the relative fitness differences among individuals along some phenotypic or external (e.g., environmental or food type) gradient, which provides the basis for natural selection [89]. These calculations are then said to provide null, testable hypotheses. Such a species-agnostic, general theoretical backbone has motivated decades of life history research across vastly different systems in a systematic manner [83,90,91]. As a famous example, reduced adult survival was predicted to drive evolution towards earlier maturation and increased reproductive effort in abstract theory, which was repeatedly supported empirically in Trinidadian guppies [90,92]. The philosophy of such theoretical fields is not to precisely explain every system with one model but to provide a common-language framework to be flexibly parameterized and tested by researchers to study their specific systems.

Similar to the phenotype-to-fitness mapping considered by life history and demography theorists, timing of occurrence or trait expression within seasonal windows is an axis that covaries with fitness [4,93,94]. The key practical benefits for phenology that these neighboring disciplines offer might be quantitative tools to deal with temporal contingencies within and across seasonal time windows. Namely, life history models specify temporal contingency in 2 main forms: (1) an individual organism’s current allocations into biological functions influence its own future allocations; and (2) current biological allocations have rippling consequences for future generations [82,83]. Thus, selection on phenological timing within one seasonal window depends on selection in past and future seasonal windows (Box 2 and Fig 3; [95,96]). Demography integrates temporal contingencies in the dynamics of stage-/age-/size-/sex- structures of populations into selection dynamics. For example, fluctuating age- or stage-structures of populations, as opposed to simply population size, influence population growth trajectories, as well as calculations of optimal phenotypes [84]. Calculations of selection on life histories when such real structural complications are considered can be very different from when they are not considered [97,98]. Another real complication of natural populations that demographic theory is suited to deal with is that individuals in populations exhibit variations in phenological schedules. For example, sexes of the same species often have different courses of seasonal developmental sequences and are affected differently by change in seasonality [99]. Seasonal synchrony of sexes is important for mating or even predator swamping [100]. For Scottish red deer, climate change has induced unequal advancements of phenological traits between males and females, leading to a contraction of their seasonal breeding window [101]. Further, different life stages of a single species can be differentially shifted by climate change. For example, in yellow-bellied marmots, advancements in dates of emergence from hibernation and weaning, but not of the onset of hibernation, led to the lengthening of their growing season, and to increases in body mass, reproduction, and population size [39].

**Box 2. Life history evolution of seasonal phenologies**

Analyzing phenological traits as independent response variables oversimplifies the manner in which life cycles are structured by trade-offs and contingencies between life history traits [75,102] (Fig 3). The expression of a trait is dependent on those that occurred earlier in the season as well as in previous seasons or generations. Selection on traits therefore depends intimately on the covariance structures of holistic life history strategies [42,103,104]. Models that account for such covariance structures are typically exercises in optimization. They ask how the potential fitness benefits of a particular phenological timing such as flowering time relative to the environmental cycle is balanced out by costs on fitness through trade-offs [105]. Costs can be incurred on an individual at the current moment in the form of decreased survival, or through any lag effects on the same individual via future survival, or on the survival probability of its progeny. Life history theory asks which of all possible combinations of such interlinked traits would confer the highest fitness for the genotypic lineage in the long run and eventually invade the population.

Life history optimization studies historically assumed constant environments, but theorists recognize that stochasticity in the environment can produce very different outcomes [97,106–109]. Stochastic demography has been applied to many interesting life history and phenological questions [84]. For example, decreased predictability of the seasonal environment may induce the evolution of bet-hedging strategies, wherein risks of potentially “incorrect” life history timing are spread among individuals, maintaining multimodal or broad distributions of life history strategies in the population [64,110–112]. Further refining our understanding of variability of the environment, recent work has investigated how the strength of temporal autocorrelation in stochastic environments influences life history evolution [64,65].

However, the manner in which life history timings are shaped by nonrandom cyclical temporal structures in the environment—such as those governed by Earth’s rotation around the sun which is geophysically locked—remains much less well understood theoretically [98]. Specifically, we have limited knowledge of how structural parameters like amplitude or period of abiotic variables, beyond just how strongly those variables are autocorrelated through time, influence life history evolution. Climate change is perturbing parameters of seasonal cycles (e.g., longer growing seasons [113,114] and greater amplitudes of annual CO_2_ cycle [115]) and phenological timing around the planet. Understanding how parameters of cycles shape life history evolution will help to explain and predict continued phenological shifts under future change [42].

Above examples of studies that incorporated life history interdependencies and demographic structure into phenological analysis demonstrate that phenology is a highly eco-evolutionary process (Box 1) and would benefit from being modeled as such. For example, phenological selection shapes the individual variation of life cycle schedules within a seasonal window. The life cycle decisions made in that seasonal window have consequences on the survival and life cycles of genotypes that make it to future seasons due to intergenerational trade-offs [82]. These genotypes then shape the standing variation of traits and population structure that comprise the raw material available for selection in future windows, completing the eco-evolutionary loop. Such a demographically explicit conceptualization of phenological evolution may be one of the most promising targets of theoretical progress [36,44].

In testing phenological evolution theory using the common types of phenological data, a nuanced conceptual gap that needs to be bridged is one between how “rate” (i.e., speed of processes or number of events in a time interval; e.g., oscillation frequency) evolves and how “timing” (i.e., the occurrence of events in reference to a clock; e.g., oscillation phase) evolves. Rates are the parameters typically manipulated in demographic and life history models due to the time differential nature of dynamical systems modeling. Such models ask what happens over a fixed time step, whether that be a large step (e.g., a month) or an infinitesimally small one (e.g., $\underset{\Delta t\to 0}{\lim}\frac{\Delta x}{\Delta t}$). Conceptions of rate, such as development, force the theorist to confront the fact that all phenology-related processes require time to complete such as size growth and physiological development. For example, when a flowering event is detected, it represents the culmination of a series of upstream biological steps leading up to that point; these can be aggregated to express a rate to reach that point. Therefore, one needs to consider the correlated and sometimes antagonistic selection pressures involved prior to the detectable timing of an event. However, the actual timing of an event is often what affects intra- and interspecies interactions such as mating or predator avoidance and determines the set of environmental conditions experienced by an individual. Timing is a measurable point event that affects survival, and thus, is potentially more “visible” to selection [2]. Further, events like flowering reflect actual categorical change with a binomial property and is thus more easily measurable than rates. Likely for these reasons, data on timing dominate phenological studies (e.g., [116]). As a starting point, rate and timing are analogous in simple cases such as annual organisms that start and end their lives in a year (i.e., fast-growers mature earlier in a season). For species with more complex life histories, this conversion does not necessarily hold true. Marrying rate-based theoretical foundations with decades of existing timing data will unlock important advances in our general understanding of phenological evolution.

### How do species interactions produce and maintain diverse phenologies in the same space?

**Fig 4:**
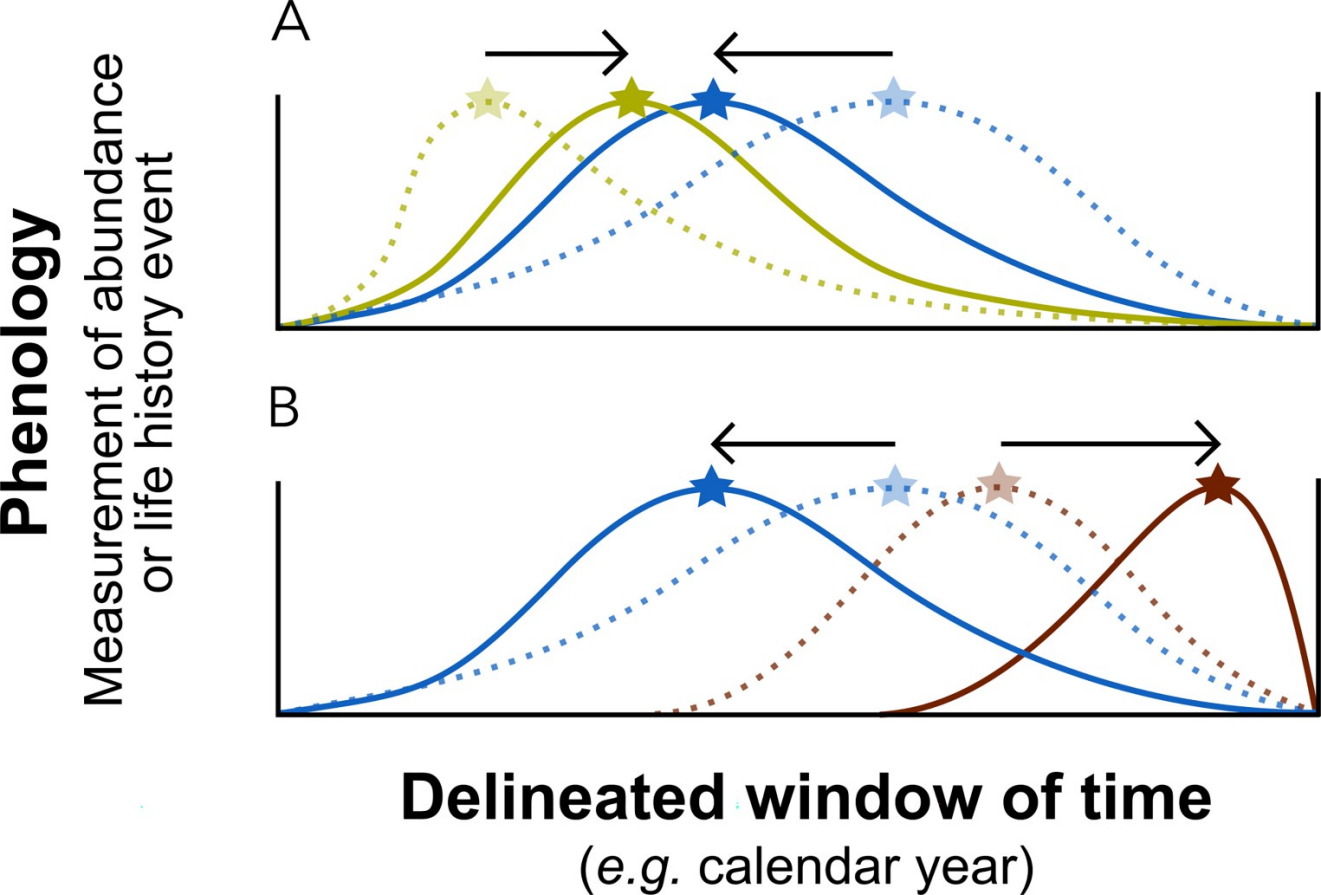
Community interactions shape phenological variation within bounded windows of time. In addition to abiotic seasonal cues and internal mechanisms of optimization at the single-species level, ecological interactions can influence the phenologies of coexisting species in a community. Colored curves show hypothetical phenological curves of species, measured as change in abundance reflecting seasonal emergence or number of individuals expressing a trait such as flowering. Stars show peak phenology. Dashed curves show phenologies prior to shifts, and arrows show direction of shifts. Certain interactions may favor (A) co-occurrence between species, such as plant–pollinator interactions and other mutualistic relationships, and others (B) avoidance, such as competition for a time-limited resource.

Phenological evolution occurs in the context of ecological communities. The challenge is to understand how periodic interactions between coexisting species influence each species’ adaptive occupation of different portions of seasonal windows. Empirical evidence shows that different types of ecological interactions such as competition, invasion, or consumer-resource dynamics can alter the occurrence or trait expression timing of species in a community. Broadly, periodic interactions can favor overlap (Fig 4A) or segregation (Fig 4B) of phenologies between 2 species within seasonal time windows. Mechanisms depend on context and history. For example, experimental reduction of plant species diversity in a serpentine grassland community in California, USA advanced the phenology of remaining species, suggesting an infilling of newly available temporal niches [117]. This suggests that competition may limit co-occurrence. Analogously, exotic plant species may invade a new community by exploiting early-season phenological niches in which competition by co-occurrence with native species is lower [118] (but see [119]). A similar pattern can be achieved through a consumer-resource dynamic: introduction of large vertebrate herbivores may have selected for advanced flowering time in forage species in the US Southwest because earlier flowering reduces herbivory-induced loss of reproductive structures [120]. Mismatches in phenological shifts across trophic levels can have adverse effects on reproduction, survival and fitness of coexisting species, and cause rapid increases in extinction probability of populations [95] or health of whole ecosystems [6]. Some trophic links such as plant–pollinator pairs appear capable of advancing constituent phenologies fairly synchronously [121,122], possibly suggesting that at least in some cases, the selective forces on phenology imposed by species interactions are dominant over those imposed by single-species life history optimization. One fruitful avenue of theoretical advancement will be to incorporate the various modes of periodic phenological interaction into models of single-species phenological evolution. Interactions can be treated as dynamic time-dependent parameters that modify fitness landscapes of each involved species. Viewing phenological communities as dynamical systems in this way might help explain many of the incongruous cases of phenological shifts that appear unintuitive when studied out of the context of the community.

The key ecological consequence of the differential expansions, contractions, and shifts among species’ phenologies is that the interaction potential between combinations of species can change within seasonal time windows [37,123,124]. Thus, novel “no-analog” communities (*sensu* [125]) can form through the season. For example, a recent 12-year observational study of 14 coexisting vascular plant species at a low-Arctic study site in Greenland revealed that differential advancement of spring emergence among the species [126] increased temporal segregation of the early- and late-phenology species from other species [29]. Among species of coexisting plants in a subalpine meadow in Colorado, USA, differential rates of advance of first, peak, and last flowering time have altered the phenological sequence and co-flowering patterns through the season [37]. Similar phenomena are now documented across a broad range of biological systems including butterflies [127], anurans [128,129], vascular plants [37,130,131], and vertebrate herbivores [29]. These cases of temporal shuffling of phenological communities highlight the issue that co-existence in the same space does not necessarily mean co-occurrence. Interaction potentials are as periodic as the occurrence of each species in seasonal systems and are being perturbed under climate change. One important question that emerges—connected to the broader disciplines of species coexistence and biodiversity research—is how perturbations to multi-phenological systems influence interaction dynamics among species within seasonal time windows and thus long-term ecological community stability and maintenance of phenological diversity (Box 3).

**Box 3. Important unresolved questions**

Within our framework of conceiving phenological phenomena as fitness-related distributions in time windows (Fig 1), we propose a set of questions for theorists and empiricists moving forward:

- To what extent are parameters of abiotic environmental cycles (e.g., the length of the climatic growing season) themselves agents of phenological selection? In other words, how do seasonal cycle parameter changes map to fitness landscape change? Does this perspective help explain phenological shifts in the context of global change, wherein parameters of environmental cycles are being altered?
- How do life history traits—which are intricately interdependent due to covariances and trade-offs—evolve upon a template of environmental cycles, which are themselves structured by temporal autocorrelation and lag effects? Recognizing connections among phenological traits, borrowing from life history theory, will advance our understanding of phenological shifts beyond correlative approaches that focus on single traits. We have highlighted that marrying concepts of timing and rate in models will be important.
- Temporal autocorrelation can occur at various resonances that may constructively or destructively interfere with seasonal cycles (e.g., monthly or multiannual cycles). How do multi-resonance regimes influence the evolution of seasonal phenology?
- Do phenologies of organisms with different numbers of generations per seasonal window evolve in fundamentally different ways, as the windows expand, contract, or become otherwise distorted (e.g., less predictable seasonal boundaries)?
- How can we better integrate empirical approaches to enhance our general understanding of phenological evolution? As an example, can models that make predictions about optimal phenology (e.g., flowering time) as a function of environmental cycle parameters (e.g., climatic growing season length) be tested by manipulating those parameters and measuring genetic and phenotypic frequency change simultaneously in experimental populations?
- How is the timing of a trait that is important from the perspective of the community (e.g., flowering) controlled simultaneously by life history optimization at the species level and periodic interactions with other species that favor co-occurrence or temporal segregation? Are there general rules regarding if and when single-species evolution or multispecies interaction is a stronger driver of phenological selection in nature?
- How do changes in seasonal time window parameters alter interaction potentials in ecological communities, create novel no-analog communities in different portions of the season, and affect phenological diversity?

### Summary and future outlook

Phenology is ubiquitous. Species around the planet have evolved a panoply of physiological, genetic, behavioral, plastic, and neuronal mechanisms to strategically utilize seasonally available time windows [4,6,41,132–135]. Our aim here was to take a broader view and ask why the seemingly universal need for these innovations exists in the first place: why exactly is timing so deeply important throughout nature?

While the sheer ubiquity of phenology itself warrants theoretical synthesis, global change makes it urgent. Perturbations to the cyclically occurring seasonal windows—analogous to spatial perturbations to habitats such as deforestation—are altering the familiar phenological patterns contained within those windows in perplexing ways [37,126]. Correlative or statistical modeling approaches that address phenological changes using candidate environmental variables yield limited lessons for a general understanding because even when the hunt for the best correlate is complete for a given system, the next system will require its own set of assumptions and candidate explanatory drivers to be tested. For phenology to be a unified eco-evolutionary discipline, the conception of fitness must move beyond qualitative or post hoc statistical justifications because those are necessarily limited to system idiosyncrasies. Much more attention needs to be given to theorizing the general timing-to-fitness map. The calculus of the map is deeply complex given the issues we have discussed. Progress can be made by explicitly integrating how populations move through iterative time windows (of changing durations under global change) based on life history decisions and demographic transitions.

The microevolutionary process of phenological evolution will be another frontier of investigation. Population genetic processes such as recombination and drift are constrained by the seasonally fluctuating probabilities of encounter among individuals, which limit periodic opportunities for mating and gene exchange, and thus the inheritance of phenological traits. Studying how all of the above processes per species are simultaneously influenced by interactions between species in ecological communities will be the next challenge. We have strived to distill key questions for future investigators, particularly those interested in developing general theory (Box 3). Empirical tests of the generality of phenological evolutionary theory will be important moving forward. Fortunately, phenological data are relatively cheap and benefit from many academic and citizen science traditions. Global scale open-access phenological databases continue to grow rapidly, such as the USA National Phenology Network and the Pan-European Phenology Database, and will make such interdisciplinary and comparative investigations possible.

Finally, the general concepts about time windows we discussed extend to a fundamental theme in ecology and evolution, beyond just the scale of seasons. While phenological research has focused on the seasonal scale, cycles in the physical environment in fact exist on many other temporal scales such as daily, tidal, and multiannual (e.g., El Niño-Southern Oscillation cycle), with “phenological” scales to match (e.g., diel vertical migration of zooplankton [136]). Geophysically driven oscillations of the environment clearly constitute a pervasive theme of temporal structure and pattern in natural systems. Scalable theory of how organisms evolve to occupy cyclical windows of ecological time, as a function of the relative scaling between those windows and the organisms’ generation times, would be a rich avenue of exploration.
